# Complete convergence and complete moment convergence for randomly weighted sums of martingale difference sequence

**DOI:** 10.1186/s13660-018-1770-3

**Published:** 2018-07-13

**Authors:** Huanhuan Ma, Yan Sun

**Affiliations:** 1 College of Mathematics and Information Science, Henan Normal University, Xinxiang, China; 2 Science College, Beijing Information Science and Technology University, Beijing, China

**Keywords:** 60F15, Martingale difference sequence, Complete convergence, Complete moment convergence, Randomly weighted sums

## Abstract

In this paper, we extend some known results about complete convergence and establish the complete convergence and complete moment convergence for randomly weighted sums of martingale difference sequence. Our results can generalize some conclusions related to Hsu–Robbins–Erdös strong laws and Baum–Katz type theorems for martingales.

## Introduction

The complete convergence plays a key role in the development of probability theory, especially in establishing the rate of convergence. Hsu and Robbins [1] introduced the concept of complete convergence as follows. A sequence $\{X_{n}, n\ge1\}$ is said to converge completely to *C* if

1.1 $$ \sum_{n=1}^{\infty}P\bigl( \vert X_{n}-C \vert \ge\epsilon\bigr)< \infty\quad \text{for all } \epsilon>0, $$

where *C* is a constant. By the Borel–Cantelli lemma, it follows that $X_{n}\to C$ almost surely as $n\to\infty$. If $\{X_{n}, n\ge1\}$ is independent and identically distributed (i.i.d.) random variables, the converse is true.

Suppose that $\{X_{n}, n\ge1\}$ is a random variable sequence defined on the fixed probability space $(\Omega, \mathcal{F}, P)$. Denote $S_{n}=\sum_{i=1}^{n}X_{i}$, $S_{0}=0$, $\log x=\log(\max\{e, x\})$, $x^{+}=xI(x\ge 0)$, and $\mathcal{F}_{0}=\{\Omega, \emptyset\}$. Let $\{\mathcal {F}_{n}, n\ge1\}$ be an increasing sequence of *σ* fields with $\mathcal{F}_{n}\subset\mathcal{F}$ for each $n\ge1$. If $X_{n}$ is $\mathcal{F}_{n}$ measurable for each $n\ge1$, then *σ* fields $\{ \mathcal{F}_{n}, n\ge1\}$ are thought to be adapted to the random variable sequence $\{X_{n}, n\ge1\}$ and $\{X_{n}, \mathcal{F}_{n}, n\ge1\} $ is thought to be an adapted stochastic sequence. The following theorem is a generalization of some known results.

### Theorem 1.1

(Hsu–Robbins–Erdös strong law [1, 2])

*Let* $\{X_{n}, n\ge1\}$ *be a sequence of independent and identically distributed random variables*. *Assume that* $EX_{n}=0$ *and set* $S_{n}=\sum_{i=1}^{n}X_{i}$, $n\ge1$. *Then* $EX_{n}^{2}<\infty$ *is equivalent to the condition that*

1.2 $$ \sum_{n=1}^{\infty}P\bigl(|S_{n}|\ge\epsilon n\bigr)< \infty\quad \textit{for all } \epsilon>0. $$

In probability theory, Hsu–Robbins–Erdös strong law as a basic theorem has been extended in several directions by some authors. The following theorem is given by Baum and Katz [3] to establish a rate of convergence.

### Theorem 1.2

(Baum and Katz strong law)

*Let* $\alpha>1/2$, $\alpha p>1$, *and let* $\{X_{n}, n\ge1\}$ *be a sequence of independent and identically distributed random variables*. *Assume that* $EX_{n}=0$ *if* $\alpha\le1$, *and set* $S_{n}=\sum_{i=1}^{n}X_{i}$, $n\ge1$. *Then* $E|X_{n}|^{p}<\infty$ *is equivalent to the condition that*

1.3 $$ \sum_{n=1}^{\infty}n^{\alpha p-2}P\bigl(|S_{n}|\ge\epsilon n^{\alpha}\bigr)< \infty \quad \textit{for all } \epsilon>0 $$

*and also equivalent to the condition that*

1.4 $$ \sum_{n=1}^{\infty}n^{\alpha p-2}P\Bigl(\max_{1\le k\le n}|S_{k}|\ge\epsilon n^{\alpha}\Bigr)< \infty\quad \textit{for all } \epsilon>0. $$

Motivated by the above results for i.i.d. random variables, many authors have studied them for dependent cases. The case for weighted sums of extended negatively dependent (END) random variable sequence was investigated by Shen et al. [4]. Miao et al. [5] improved some known results and studied the Baum–Katz type convergence rate in the Marcinkiewicz–Zygmund strong law for martingales. Chen et al. [6] also gave some extended results for the sequence of martingale difference.

The aims of the present paper are to extend the results on complete convergence for the sequence of martingale difference. The following definitions will be used frequently in this paper.

### Definition 1.1

If $\{X_{n}, \mathcal{F}_{n}, n\ge1\}$ is an adapted stochastic sequence with

$$E(X_{n}|\mathcal{F}_{n-1})=0 \quad \mbox{a.s.} $$

and $E|X_{n}|<\infty$ for each $n\ge1$, then the sequence $\{X_{n}, \mathcal{F}_{n}, n\ge1\}$ is called a martingale difference sequence.

### Definition 1.2

A real-valued function $l(x)$, positive and measurable on $(0, \infty )$, is said to be slowly varying if

$$\lim_{x\to\infty}\frac{l(\lambda x)}{l(x)}=1 $$

for each $\lambda>0$.

### Definition 1.3

A sequence $\{X_{n}, n\ge1\}$ of random variables is said to be stochastically dominated by a random variable *X* if there exists a positive constant *C*, such that

$$P\bigl( \vert X_{n} \vert >x\bigr)\le CP\bigl( \vert X \vert >x\bigr) $$

for all $x\ge0$ and $n\ge1$.

Now let us recall some known results for complete convergence of martingales.

### Theorem 1.3

([7, 8])

*Let* $\{X_{n}, \mathcal{F}_{n}, n\ge1\}$ *be an* $L^{p}$-*bounded martingale difference sequence*. *If* $0<1/\alpha<2<p$ *or* $1< p<2$, $1\le1/\alpha\le p$, *then*

1.5 $$ \sum_{n=1}^{\infty}n^{\alpha p-2}P\bigl(|S_{n}|\ge\epsilon n^{\alpha}\bigr)< \infty \quad \textit{for all } \epsilon>0. $$

*If* $p=\alpha=1$, *the martingale difference sequence satisfies*

$$\sup_{n\ge1}E|X_{n}|\log|X_{n}|< \infty, $$

*then* (1.5) *holds*.

Wang and Hu [9] further studied the Baum–Katz type theorem for the maximal partial sum of martingale difference sequence.

### Theorem 1.4

[9] *Let* $\{X_{n}, \mathcal{F}_{n}, n\ge1\}$ *be a sequence of martingale difference*, *which is stochastically dominated by a random variable* *X*. *Let* $l(x)>0$ *be a slowly varying function as* $x\to\infty$. *Let* $\alpha>1/2$, $p\ge1$ *and* $\alpha p\ge1$. *When* $p\ge2$, *we further assume that*

$$E\Bigl[\sup_{i\ge1}E\bigl(X_{i}^{2}| \mathcal{F}_{i-1}\bigr)\Bigr]^{q/2}< \infty $$

*for some* $q>\frac{2(\alpha p-1)}{2\alpha-1}$. *If*

1.6 $$ E|X|^{p}l\bigl(|X|^{1/\alpha}\bigr)< \infty, $$

*then for any* $\epsilon>0$,

1.7 $$ \sum_{n=1}^{\infty}n^{\alpha p-2}l(n)P\Bigl(\max_{1\le j\le n}|S_{j}|\ge \epsilon n^{\alpha}\Bigr)< \infty. $$

Yang et al. [10] generalized the results of Stoica [7, 8] and Wang et al. [11] for the nonweighted sums of martingale difference sequence to the case of randomly weighted sums.

### Theorem 1.5

[10] *Let* $\{X_{n}, \mathcal{F}_{n}, n\ge1\}$ *be a martingale difference sequence stochastically dominated by a nonnegative random variable* *X* *with* $EX^{p}<\infty$. *Assume that* $\{A_{n}, n\ge1\}$ *is a random sequence*, *and it is independent of* $\{X_{n}, n\ge1\}$. *Denote* $\mathcal {G}_{0}=\{\emptyset, \Omega\}$ *and* $\mathcal{G}_{n}=\sigma(X_{1},\ldots, X_{n})$, $n\ge1$. *Let* $\alpha>1/2$, $1< p<2$, *and* $1\le\alpha<2$. *If*

1.8 $$ \sum_{i=1}^{n}EA_{i}^{2}=O(n), $$

*then*

1.9 $$ \sum_{n=1}^{\infty}n^{\alpha p-2-\alpha}E \Biggl(\max_{1\le k\le n} \Biggl\vert \sum _{i=1}^{n}A_{i}X_{i} \Biggr\vert -\epsilon n^{\alpha}\Biggr)^{+}< \infty\quad \textit{for any } \epsilon>0. $$

*If* $\alpha>1/2$, $p\ge2$, *and for some* $q>\frac{2(\alpha p-1)}{2\alpha -1}$, *we assume that*

$$E\Bigl[\sup_{i\ge1}E\bigl(X_{i}^{2}| \mathcal{G}_{i-1}\bigr)\Bigr]^{q/2}< \infty. $$

*Let*

1.10 $$ \sum_{i=1}^{n}E|A_{i}|^{q}=O(n), $$

*then* (1.9) *holds*.

*If* $\alpha>0$ *and* $p=1$, *the martingale difference sequence is stochastically dominated by a nonnegative random variable* *X* *with* $E[X\log(1+X)]<\infty$, *and* (1.8) *holds*, *then*

1.11 $$ \sum_{n=1}^{\infty}n^{-2}E \Biggl(\max_{1\le k\le n} \Biggl\vert \sum _{i=1}^{n}A_{i}X_{i} \Biggr\vert -\epsilon n^{\alpha}\Biggr)^{+}< \infty \quad \textit{for any } \epsilon>0. $$

We shall study the complete convergence and complete moment convergence for randomly weighted sums of martingale difference sequence. The paper is organized as follows. The next section is devoted to the descriptions of our main results, and their proofs will be given in Sect. 3. Throughout the paper, we use the constant *C* to denote a universal real number that is not necessarily the same in each appearance.

## Main results

### Theorem 2.1

*Let* $\{X_{n}, \mathcal{F}_{n}, n\ge1\}$ *be a sequence of martingale difference*, *which is stochastically dominated by a random variable* *X*. *Let* $l(x)>0$ *be a slowly varying function as* $x\to\infty$. *Suppose that* $\{b_{n}, n\ge1\}$ *and* $\{c_{n}, n\ge1\}$ *are sequences of positive constants such that*, *for* $p>1$, $\alpha>0$, $\alpha p\ge1$, *and some* $q\ge\max\{2, p\}$,

2.1 $$ \sum_{n=1}^{m} \frac{nb_{n}l(n)}{c_{n}}=O \bigl(c_{m}^{p-1}l\bigl(c_{m}^{1/\alpha } \bigr) \bigr), \qquad \sum_{n=m}^{\infty}\frac{nb_{n}l(n)}{c_{n}^{q}}=O \bigl(c_{m}^{p-q}l\bigl(c_{m}^{1/\alpha} \bigr) \bigr), $$

*and*

2.2 $$ \sum_{n=1}^{\infty}\frac{n^{\frac{q}{2}}b_{n}l(n)}{c_{n}^{q}}< \infty,\qquad E \Bigl[\sup_{i\ge1}E \bigl(X_{i}^{2}|\mathcal{G}_{i-1} \bigr) \Bigr]^{\frac{q}{2}}< \infty, $$

*where* $c_{n}\to\infty$ *as* $n\to\infty$. *Assume that* $\{A_{n}, n\ge1\}$ *is a random sequence independent of* $\{ X_{n}, n\ge1\}$ *such that*

2.3 $$ \sum_{i=1}^{n}E|A_{i}|^{q}=O(n). $$

*If*

2.4 $$ E|X|^{p}l\bigl(|X|^{1/\alpha}\bigr)< \infty, $$

*then for any* $\epsilon>0$,

2.5 $$ \sum_{n=1}^{\infty}b_{n}l(n)P \Biggl(\max_{1\le j\le n} \Biggl\vert \sum _{i=1}^{j}A_{i}X_{i} \Biggr\vert \ge\epsilon c_{n} \Biggr)< \infty. $$

### Corollary 2.1

*Under the conditions of Theorem *2.1, *we take* $b_{n}=n^{\alpha p-2}$, $c_{n}=n^{\alpha}$ *for* $\alpha>1/2$, $p>1$, *and* $\alpha p\ge1$. *If*

$$\textstyle\begin{cases} {q>\max \{p, \frac{2(\alpha p-1)}{2\alpha-1} \}},&p\ge 2; \\ {q=2},& 1< p< 2; \end{cases} $$

*then for any* $\epsilon>0$,

2.6 $$ \sum_{n=1}^{\infty}n^{\alpha p-2}l(n)P \Biggl(\max_{1\le j\le n} \Biggl\vert \sum _{i=1}^{j}A_{i}X_{i} \Biggr\vert \ge\epsilon n^{\alpha}\Biggr)< \infty. $$

### Remark 2.1

Obviously, (2.6) can be checked by Theorem 2.1 and Lemma 3.4. Under the conditions of Corollary 2.1, if we take $A_{i}\equiv1$, $i\ge1$, then we have (1.7), i.e., the conclusion of Wang and Hu [9] holds for $p>1$. On the other hand, if we take $l(x)\equiv1$, then we can get Remark 2.1 in Yang et al. [10]. So our results can imply these known results.

### Example 2.1

Under the conditions of Theorem 2.1, we take $b_{n}=n^{r-2}$, $l(n)=\log n$, and $c_{n}=n^{r/p}$ for $p>1$ and $r>p$. If

$$\textstyle\begin{cases} {q>\max \{p, \frac{2p(1-r)}{p-2r} \}},&p\ge2; \\ {q=2},& 1< p< 2; \end{cases} $$

then for any $\epsilon>0$,

$$\sum_{n=1}^{\infty}n^{r-2}\log nP \Biggl(\max_{1\le j\le n} \Biggl\vert \sum _{i=1}^{j}A_{i}X_{i} \Biggr\vert \ge\epsilon n^{r/p} \Biggr)< \infty. $$

### Example 2.2

Under the conditions of Theorem 2.1, we take $b_{n}=\frac{\log n}{n}$, $l(n)=\log n$, and $c_{n}=(n\log n)^{\frac{1}{p}}$ for $1< p\le2$. If

$$\textstyle\begin{cases} {q>6},&p=2; \\ {q=2},&1< p< 2; \end{cases} $$

then for any $\epsilon>0$,

$$\sum_{n=1}^{\infty}\frac{(\log n)^{2}}{n}P \Biggl( \max_{1\le j\le n} \Biggl\vert \sum_{i=1}^{j}A_{i}X_{i} \Biggr\vert \ge\epsilon(n\log n)^{\frac {1}{p}} \Biggr)< \infty. $$

### Theorem 2.2

*Let* $\{X_{n}, \mathcal{F}_{n}, n\ge1\}$ *be a sequence of martingale difference*, *which is stochastically dominated by a random variable* *X*. *Let* $l(x)>0$ *be a slowly varying function as* $x\to\infty$. *Suppose that* $\{b_{n}, n\ge1\}$ *and* $\{c_{n}, n\ge1\}$ *are sequences of positive constants such that*, *for* $p>1$, $\alpha>0$, $\alpha p\ge1$, *and some* $q\ge\max\{2, p\}$,

2.7 $$ \sum_{n=1}^{m}nb_{n}l(n)=O \bigl(c_{m}^{p-1}l\bigl(c_{m}^{1/\alpha}\bigr) \bigr),\qquad \sum_{n=m}^{\infty}\frac{nb_{n}l(n)}{c_{n}^{q-1}}=O \bigl(c_{m}^{p-q}l\bigl(c_{m}^{1/\alpha} \bigr) \bigr), $$

*and*

2.8 $$ \sum_{n=1}^{\infty}\frac{n^{\frac{q}{2}}b_{n}l(n)}{c_{n}^{q-1}}< \infty,\qquad E \Bigl[\sup_{i\ge1}E \bigl(X_{i}^{2}|\mathcal{G}_{i-1} \bigr) \Bigr]^{\frac{q}{2}}< \infty, $$

*where* $c_{n}\to\infty$ *as* $n\to\infty$. *Assume that* $\{A_{n}, n\ge1\}$ *is a random sequence independent of* $\{ X_{n}, n\ge1\}$ *such that*

2.9 $$ \sum_{i=1}^{n}E|A_{i}|^{q}=O(n). $$

*If*

2.10 $$ E|X|^{p}l\bigl(|X|^{1/\alpha}\bigr)< \infty, $$

*then for any* $\epsilon>0$,

2.11 $$ \sum_{n=1}^{\infty}b_{n}l(n)E \Biggl(\max_{1\le j\le n} \Biggl\vert \sum _{i=1}^{j}A_{i}X_{i} \Biggr\vert -\epsilon c_{n} \Biggr)^{+}< \infty. $$

### Theorem 2.3

*Let* $\{X_{n}, \mathcal{F}_{n}, n\ge1\}$ *be a sequence of martingale difference*, *which is stochastically dominated by a random variable* *X*. *Let* $l(x)>0$ *be a slowly varying function as* $x\to\infty$. *Suppose that* $\{b_{n}, n\ge1\}$ *and* $\{c_{n}, n\ge1\}$ *are sequences of positive constants such that*, *for* $p=1$, $\alpha>0$, $\alpha p\ge1$, *and some* $q\ge2$,

2.12 $$ \sum_{n=1}^{m}nb_{n}l(n)=O \bigl((\log c_{m})l\bigl(c_{m}^{1/\alpha}\bigr) \bigr), \qquad \sum_{n=m}^{\infty}\frac{nb_{n}l(n)}{c_{n}}=O \bigl(c_{m}^{1-q}l\bigl(c_{m}^{1/\alpha}\bigr) \bigr), $$

*where* $c_{n}\to\infty$ *as* $n\to\infty$. *Assume that* $\{A_{n}, n\ge1\}$ *is a random sequence independent of* $\{ X_{n}, n\ge1\}$ *and satisfying* (2.9). *If*

2.13 $$ E \bigl[ \vert X \vert \bigl(\log \vert X \vert \bigr)l \bigl( \vert X \vert ^{1/\alpha}\bigr) \bigr]< \infty, $$

*then we have formula* (2.11).

### Corollary 2.2

*Under the conditions of Theorem *2.2 *for* $p>1$, *we take* $b_{n}=n^{\alpha p-2-\alpha}$, $c_{n}=n^{\alpha}$ *for* $\alpha>1/2$ *and* $\alpha p\ge1$. *If*

$$\textstyle\begin{cases} {q>\max \{p, \frac{2(\alpha p-1)}{2\alpha-1} \}},&p\ge 2;\\ {q=2},& 1< p< 2; \end{cases} $$

*then for any* $\epsilon>0$,

2.14 $$ \sum_{n=1}^{\infty}n^{\alpha p-2-\alpha}l(n)E \Biggl(\max_{1\le j\le n} \Biggl\vert \sum _{i=1}^{j}A_{i}X_{i} \Biggr\vert -\epsilon n^{\alpha}\Biggr)^{+}< \infty. $$

*Under the conditions of Theorem *2.3 *for* $p=1$, *if we take* $b_{n}=n^{-2}$, $c_{n}=n^{\alpha}$ *for* $\alpha>0$. *Then*, *for any* $\epsilon >0$, *we have*

2.15 $$ \sum_{n=1}^{\infty}n^{-2}l(n)E \Biggl(\max_{1\le j\le n} \Biggl\vert \sum _{i=1}^{j}A_{i}X_{i} \Biggr\vert -\epsilon n^{\alpha}\Biggr)^{+}< \infty. $$

### Remark 2.2

Obviously, (2.14) and (2.15) can be checked by Theorem 2.2, Theorem 2.3, and Lemma 3.4. Under the conditions of Corollary 2.2, if we take $l(x)\equiv1$, then we have (1.9) and (1.11), i.e., the results of Yang et al. [10] can be generalized by our conclusions. On the other hand, if we take $A_{i}\equiv1$, $i\ge1$, then we can get Theorem 3.3 and Theorem 3.4 in Wang and Hu [9]. Hence, our conclusions can extend these known results.

### Example 2.3

Under the conditions of Theorem 2.2, we take $b_{n}=n^{r-2-r/p}$, $l(n)=\log n$, and $c_{n}=n^{r/p}$ for $p>1$ and $r>p$. If

$$\textstyle\begin{cases} {q>\max \{p, \frac{2p(1-r)}{p-2r} \}},&p\ge2;\\ {q=2},& 1< p< 2; \end{cases} $$

then for any $\epsilon>0$,

$$\sum_{n=1}^{\infty}n^{r-2-r/p}\log nE \Biggl(\max_{1\le j\le n} \Biggl\vert \sum _{i=1}^{j}A_{i}X_{i} \Biggr\vert -\epsilon n^{r/p} \Biggr)^{+}< \infty. $$

### Example 2.4

Under the conditions of Theorem 2.2, we take $b_{n}=\frac{(\log n)^{1-1/p}}{n^{1+1/p}}$, $l(n)= (\log n)^{1-1/p}$ and $c_{n}=(n\log n)^{\frac {1}{p}}$ for $1< p\le2$. If

$$\textstyle\begin{cases} {q>5},&p=2;\\ {q=2},&1< p< 2; \end{cases} $$

then for any $\epsilon>0$,

$$\sum_{n=1}^{\infty}\frac{(\log n)^{2-2/p}}{n^{1+1/p}}E \Biggl( \max_{1\le j\le n} \Biggl\vert \sum_{i=1}^{j}A_{i}X_{i} \Biggr\vert -\epsilon(n\log n)^{\frac{1}{p}} \Biggr)^{+}< \infty. $$

### Remark 2.3

If the conditions of Theorem 2.2 or Theorem 2.3 hold, then for any $\epsilon>0$, we can get

$$\sum_{n=1}^{\infty}b_{n}c_{n}l(n)P \Biggl(\max_{1\le j\le n} \Biggl\vert \sum _{i=1}^{j}A_{i}X_{i} \Biggr\vert \ge\epsilon c_{n} \Biggr)< \infty. $$

In fact, it can be checked that for any $\epsilon>0$,

$$\begin{aligned}& \sum_{n=1}^{\infty}b_{n}l(n)E \Biggl(\max_{1\le j\le n} \Biggl\vert \sum _{i=1}^{j}A_{i}X_{i} \Biggr\vert -\epsilon c_{n} \Biggr)^{+} \\& \quad = \sum_{n=1}^{\infty}b_{n}l(n) \int_{0}^{\infty}P \Biggl(\max_{1\le j\le n} \Biggl\vert \sum_{i=1}^{j}A_{i}X_{i} \Biggr\vert -\epsilon c_{n}>t \Biggr)\,dt \\& \quad \ge \sum_{n=1}^{\infty}b_{n}l(n) \int_{0}^{\epsilon c_{n}}P \Biggl(\max_{1\le j\le n} \Biggl\vert \sum_{i=1}^{j}A_{i}X_{i} \Biggr\vert -\epsilon c_{n}>t \Biggr)\,dt \\& \quad \ge \epsilon\sum_{n=1}^{\infty}b_{n}c_{n}l(n)P \Biggl(\max_{1\le j\le n} \Biggl\vert \sum_{i=1}^{j}A_{i}X_{i} \Biggr\vert \ge2\epsilon c_{n} \Biggr). \end{aligned}$$

### Remark 2.4

If $A_{n}=a_{n}$, $n\ge1$ is non-random (the case of constant weighted), then we can get the results of Theorems 2.1–2.3 for the non-random weighted sums of martingale difference sequence.

## Proofs for the main results

Throughout this section, we use the constant *C* to denote a generic real number that is not necessarily the same in each appearance.

### Several lemmas

To prove the main results of the paper, we need to recall the following lemmas.

#### Lemma 3.1

([12])

*If* $\{X_{i}, \mathcal{F}_{i}, 1\le i\le n\}$ *is a sequence of martingale difference and* $q>0$, *then there exists a constant* *C* *depending only on* *p* *such that*

$$E \Biggl(\max_{1\le k\le n} \Biggl\vert \sum _{i=1}^{k}X_{i} \Biggr\vert ^{q} \Biggr)\le C \Biggl\{ E \Biggl(\sum_{i=1}^{n}E \bigl(X_{i}^{2}|\mathcal{F}_{i-1}\bigr) \Biggr)^{q/2}+E \Bigl(\max_{1\le i\le n}|X_{i}|^{q} \Bigr) \Biggr\} . $$

#### Lemma 3.2

([13–15])

*Let* $\{X_{n}, n\ge1\}$ *be a sequence of random variables*, *which is stochastically dominated by a random variable* *X*. *Then*, *for any* $a>0$ *and* $b>0$, *the following two statements hold*:

$$E\bigl[ \vert X_{n} \vert ^{a}I\bigl( \vert X_{n} \vert \le b\bigr)\bigr]\le CE\bigl[ \vert X \vert ^{a}I\bigl( \vert X \vert \le b\bigr)\bigr]+b^{a}P\bigl( \vert X \vert >b\bigr) $$

*and*

$$E\bigl[ \vert X_{n} \vert ^{a}I\bigl( \vert X_{n} \vert > b\bigr)\bigr]\le CE\bigl[ \vert X \vert ^{a}I\bigl( \vert X \vert > b\bigr)\bigr]. $$

#### Lemma 3.3

[16] *Let* $\{Y_{n}, n\ge1\}$ *and* $\{Z_{n}, n\ge1\}$ *be sequences of random variables*. *Then*, *for any* $q>1$, $\epsilon>0$, *and* $a>0$,

$$\begin{aligned} E \Biggl(\max_{1\le k\le n} \Biggl\vert \sum _{i=1}^{k}(Y_{i}+Z_{i}) \Biggr\vert -\epsilon a \Biggr)^{+} \le& \biggl(\frac{1}{\epsilon^{q}}+\frac {1}{q-1} \biggr)\frac{1}{a^{q-1}}E \Biggl(\max_{1\le k\le n} \Biggl\vert \sum _{i=1}^{k}Y_{i} \Biggr\vert ^{q} \Biggr) \\ &{}+E \Biggl(\max_{1\le k\le n} \Biggl\vert \sum _{i=1}^{k}Z_{i} \Biggr\vert \Biggr). \end{aligned}$$

#### Lemma 3.4

([17])

*If* $l(x)>0$ *is a slowly varying function as* $x\to\infty$, *then*

1. $\lim_{x\to\infty}\frac{l(tx)}{l(x)}=1$ *for each* $t>0$; $\lim_{x\to\infty}\frac{l(x+u)}{l(x)}=1$ *for each* $u>0$;
2. $\lim_{k\to\infty}\sup_{2^{k}\le x<2^{k+1}}\frac {l(x)}{l(2^{k})}=1$;
3. $\lim_{x\to\infty}x^{\delta}l(x)=\infty$, $\lim_{x\to\infty }x^{-\delta} l(x)=0$ *for each* $\delta>0$;
4. $C_{1}2^{kr}l(\epsilon2^{k})\le\sum_{j=1}^{k}2^{jr}l(\epsilon2^{j})\le C_{2}2^{kr}l(\epsilon2^{k})$ *for every* $r>0$, $\epsilon>0$, *positive integer* *k*, *and some* $C_{1}>0$, $C_{2}>0$;
5. $C_{1}2^{kr}l(\epsilon2^{k})\le\sum_{j=k}^{\infty}2^{jr}l(\epsilon 2^{j})\le C_{2}2^{kr}l(\epsilon2^{k})$ *for every* $r<0$, $\epsilon>0$, *positive integer* *k*, *and some* $C_{1}>0$, $C_{2}>0$.

### Proof of Theorem 2.1

For fixed $n\ge1$, denote

$$Y_{ni}=A_{i}X_{i}I\bigl( \vert X_{i} \vert \le c_{n}\bigr)-E\bigl[A_{i}X_{i}I \bigl( \vert X_{i} \vert \le c_{n}\bigr)|\mathcal {G}_{i-1}\bigr],\quad i=1, 2, \ldots. $$

Since

$$A_{i}X_{i}=A_{i}X_{i}I\bigl( \vert X_{i} \vert >c_{n}\bigr)+Y_{ni}+E \bigl[A_{i}X_{i}I\bigl( \vert X_{i} \vert \le c_{n}\bigr)|\mathcal{G}_{i-1}\bigr], $$

we have

3.1 $$\begin{aligned}& \sum_{n=1}^{\infty}b_{n}l(n)P \Biggl(\max_{1\le j\le n} \Biggl\vert \sum _{i=1}^{j}A_{i}X_{i} \Biggr\vert \ge\epsilon c_{n} \Biggr) \\& \quad \le \sum_{n=1}^{\infty}b_{n}l(n)P \Biggl(\max_{1\le j\le n} \Biggl\vert \sum _{i=1}^{j}A_{i}X_{i}I \bigl( \vert X_{i} \vert >c_{n}\bigr) \Biggr\vert \ge \epsilon c_{n}/3 \Biggr) \\& \qquad {} +\sum_{n=1}^{\infty}b_{n}l(n)P \Biggl(\max_{1\le j\le n} \Biggl\vert \sum _{i=1}^{j}E\bigl[A_{i}X_{i}I \bigl( \vert X_{i} \vert \le c_{n}\bigr)| \mathcal{G}_{i-1}\bigr] \Biggr\vert \ge\epsilon c_{n}/3 \Biggr) \\& \qquad {} +\sum_{n=1}^{\infty}b_{n}l(n)P \Biggl(\max_{1\le j\le n} \Biggl\vert \sum _{i=1}^{j}Y_{ni} \Biggr\vert \ge \epsilon c_{n}/3 \Biggr) \\& \quad = :H+I+J. \end{aligned}$$

To prove (2.5), it is enough to show $H<\infty, I<\infty$, and $J<\infty$. Obviously, it follows from Hölder’s inequality, Lyapunov’s inequality, and (2.3) that

3.2 $$ \sum_{i=1}^{n}E|A_{i}| \le \Biggl(\sum_{i=1}^{n}E|A_{i}|^{q} \Biggr)^{\frac {1}{q}} \Biggl(\sum_{i=1}^{n}1 \Biggr)^{1-\frac{1}{q}}=O(n). $$

By the fact that $\{A_{n}, n\ge1\}$ is independent of $\{X_{n}, n\ge1\}$, it is easy to check by Markov’s inequality, Lemma 3.2, (3.2), (2.1), and (2.4) that

3.3 $$\begin{aligned} H \le& C\sum_{n=1}^{\infty}\frac{b_{n}l(n)}{c_{n}}E \Biggl(\max_{1\le j\le n} \Biggl\vert \sum _{i=1}^{j}A_{i}X_{i}I\bigl( \vert X_{i} \vert >c_{n}\bigr) \Biggr\vert \Biggr) \\ \le& C\sum_{n=1}^{\infty}\frac{b_{n}l(n)}{c_{n}} \sum_{i=1}^{n}E \vert A_{i} \vert E\bigl[ \vert X_{i} \vert I\bigl( \vert X_{i} \vert >c_{n}\bigr)\bigr] \\ \le& C\sum_{n=1}^{\infty}\frac{nb_{n}l(n)}{c_{n}}E \bigl[ \vert X \vert I\bigl( \vert X \vert >c_{n}\bigr)\bigr] \\ =& C\sum_{n=1}^{\infty}\frac{nb_{n}l(n)}{c_{n}}\sum _{m=n}^{\infty}E\bigl[ \vert X \vert I \bigl(c_{m}< \vert X \vert \le c_{m+1}\bigr)\bigr] \\ =& C\sum_{m=1}^{\infty}E\bigl[ \vert X \vert I\bigl(c_{m}< \vert X \vert \le c_{m+1}\bigr)\bigr] \sum_{n=1}^{m}\frac {nb_{n}l(n)}{c_{n}} \\ \le& C\sum_{m=1}^{\infty}E\bigl[ \vert X \vert I\bigl(c_{m}< \vert X \vert \le c_{m+1}\bigr) \bigr]c_{m}^{p-1}l\bigl(c_{m}^{1/\alpha}\bigr) \\ \le& CE \vert X \vert ^{p}l\bigl( \vert X \vert ^{1/\alpha}\bigr)< \infty. \end{aligned}$$

For *I*, since $\{X_{n}, \mathcal{F}_{n}, n\ge1\}$ is a sequence of martingale difference, we can see that $\{X_{n}, \mathcal{G}_{n}, n\ge1\} $ is also a sequence of martingale difference. Combining with the fact that $\{A_{n}, n\ge1\}$ is independent of $\{X_{n}, n\ge1\}$, we have

$$\begin{aligned} E(A_{n}X_{n}|\mathcal{G}_{n-1})&=E \bigl[E(A_{n}X_{n}|\mathcal{G}_{n})|\mathcal {G}_{n-1}\bigr] \\ &=E\bigl[X_{n}E(A_{n}|\mathcal{G}_{n})| \mathcal{G}_{n-1}\bigr] \\ &=EA_{n}E(X_{n}|\mathcal{G}_{n-1}) \\ &=0\quad \mbox{a.s.}, n\ge1. \end{aligned}$$

Consequently, by Markov’s inequality and the proof of (3.3), we have

3.4 $$\begin{aligned} I \le&C\sum_{n=1}^{\infty}\frac{b_{n}l(n)}{c_{n}}E \Biggl(\max_{1\le j\le n} \Biggl\vert \sum _{i=1}^{j}E\bigl[A_{i}X_{i}I \bigl( \vert X_{i} \vert \le c_{n}\bigr)| \mathcal{G}_{i-1}\bigr] \Biggr\vert \Biggr) \\ \le&C\sum_{n=1}^{\infty}\frac{b_{n}l(n)}{c_{n}}E \Biggl(\max_{1\le j\le n} \Biggl\vert \sum _{i=1}^{j}E\bigl[A_{i}X_{i}I \bigl( \vert X_{i} \vert >c_{n}\bigr)| \mathcal{G}_{i-1}\bigr] \Biggr\vert \Biggr) \\ \le&C\sum_{n=1}^{\infty}\frac{b_{n}l(n)}{c_{n}}\sum _{i=1}^{n}E \vert A_{i} \vert E\bigl[ \vert X_{i} \vert I\bigl( \vert X_{i} \vert >c_{n}\bigr)\bigr] \\ \le&CE \vert X \vert ^{p}l\bigl( \vert X \vert ^{1/\alpha}\bigr)< \infty. \end{aligned}$$

Next, we shall show that $J<\infty$. Let $X_{ni}=X_{i}I(|X_{i}|\le c_{n})$ and $\hat{Y}_{ni}=a_{i}X_{ni}-E(a_{i}X_{ni}|\mathcal{G}_{i-1})$. It can be found that for fixed real numbers $a_{1}, \ldots, a_{n}$, $\{\hat {Y}_{ni}, \mathcal{G}_{i}, 1\le i\le n\}$ is also a sequence of martingale difference. Note that $\{A_{1}, \ldots, A_{n}\}$ is independent of $\{X_{n1}, \ldots, X_{nn}\}$. So, by Markov’s inequality and Lemma 3.1, we have

3.5 $$\begin{aligned} J \le&C\sum_{n=1}^{\infty}\frac{b_{n}l(n)}{c_{n}^{q}}E \Biggl(\max_{1\le j\le n} \Biggl\vert \sum _{i=1}^{j}Y_{ni} \Biggr\vert \Biggr)^{q} \\ =&C\sum_{n=1}^{\infty}\frac{b_{n}l(n)}{c_{n}^{q}}E \Biggl\{ E \Biggl(\max_{1\le j\le n} \Biggl\vert \sum _{i=1}^{j}\bigl[a_{i}X_{ni}-E(a_{i}X_{ni}| \mathcal {G}_{i-1})\bigr] \Biggr\vert ^{q} \Biggr) \Big|A_{1}=a_{1}, \ldots, A_{n}=a_{n} \Biggr\} \\ \le&C\sum_{n=1}^{\infty}\frac{b_{n}l(n)}{c_{n}^{q}}E \Biggl\{ E \Biggl(\sum_{i=1}^{n}E \bigl( \hat{Y}_{ni}^{2}|\mathcal{G}_{i-1} \bigr) \Biggr)^{q/2}+\sum_{i=1}^{n}E| \hat{Y}_{ni}|^{q} \Big|A_{1}=a_{1}, \ldots, A_{n}=a_{n} \Biggr\} \\ =&C\sum_{n=1}^{\infty}\frac{b_{n}l(n)}{c_{n}^{q}}\sum _{i=1}^{n}E|Y_{ni}|^{q}+C \sum_{n=1}^{\infty}\frac{b_{n}l(n)}{c_{n}^{q}}E \Biggl( \sum_{i=1}^{n}E \bigl(Y_{ni}^{2}| \mathcal{G}_{i-1} \bigr) \Biggr)^{q/2} \\ =:&J_{1}+J_{2}. \end{aligned}$$

For $J_{1}$, we have by $C_{r}$-inequality, Lemma 3.2 with $b=c_{n}$, and (2.3) that

3.6 $$\begin{aligned} J_{1} \le&C\sum_{n=1}^{\infty}\frac{b_{n}l(n)}{c_{n}^{q}}\sum_{i=1}^{n}E \vert A_{i} \vert ^{q}E \bigl[ \vert X_{i} \vert ^{q}I\bigl( \vert X_{i} \vert \le c_{n}\bigr) \bigr] \\ \le&C\sum_{n=1}^{\infty}\frac{b_{n}l(n)}{c_{n}^{q}}\sum _{i=1}^{n}E \vert A_{i} \vert ^{q}E \bigl[ \vert X \vert ^{q}I\bigl( \vert X \vert \le c_{n}\bigr) \bigr]+C\sum_{n=1}^{\infty}\frac{b_{n}l(n)}{c_{n}^{q}}\sum_{i=1}^{n}c_{n}^{q}P \bigl( \vert X \vert >c_{n}\bigr) \\ =&C\sum_{n=1}^{\infty}\frac{nb_{n}l(n)}{c_{n}^{q}}E \bigl[ \vert X \vert ^{q}I\bigl( \vert X \vert \le c_{n}\bigr) \bigr]+C\sum_{n=1}^{\infty}nb_{n}l(n)P\bigl( \vert X \vert >c_{n}\bigr) \\ \le&C\sum_{n=1}^{\infty}\frac{nb_{n}l(n)}{c_{n}^{q}}E \bigl[ \vert X \vert ^{q}I\bigl( \vert X \vert \le c_{n}\bigr) \bigr]+C\sum_{n=1}^{\infty}\frac{nb_{n}l(n)}{c_{n}}E\bigl[ \vert X \vert I\bigl( \vert X \vert >c_{n}\bigr)\bigr] \\ =:&CJ_{11}+CJ_{12}. \end{aligned}$$

For $J_{11}$, we have by (2.1) and (2.4) that

3.7 $$\begin{aligned} J_{11} =&\sum_{n=1}^{\infty}\frac{nb_{n}l(n)}{c_{n}^{q}}\sum_{m=1}^{n}E \bigl[ \vert X \vert ^{q}I\bigl(c_{m-1}< \vert X \vert \le c_{m}\bigr) \bigr] \\ =&\sum_{m=1}^{\infty}E \bigl[ \vert X \vert ^{q}I\bigl(c_{m-1}< \vert X \vert \le c_{m}\bigr) \bigr]\sum_{n=m}^{\infty}\frac{nb_{n}l(n)}{c_{n}^{q}} \\ \le&C\sum_{m=1}^{\infty}E \bigl[ \vert X \vert ^{q}I\bigl(c_{m-1}< \vert X \vert \le c_{m}\bigr) \bigr]c_{m}^{p-q}l\bigl(c_{m}^{1/\alpha} \bigr) \\ \le&CE \vert X \vert ^{p}l\bigl( \vert X \vert ^{1/\alpha}\bigr)< \infty. \end{aligned}$$

By the proof of (3.3), it follows

3.8 $$\begin{aligned} J_{12}&=\sum_{n=1}^{\infty}\frac{nb_{n}l(n)}{c_{n}}E\bigl[ \vert X \vert I\bigl( \vert X \vert >c_{n}\bigr)\bigr] \\ &\le CE \vert X \vert ^{p}l\bigl( \vert X \vert ^{1/\alpha}\bigr)< \infty. \end{aligned}$$

Furthermore, by Hölder’s inequality and (2.3), for any $1< p\le2$, we have

3.9 $$ \sum_{i=1}^{n}E|A_{i}|^{p} \le \Biggl(\sum_{i=1}^{n}E|A_{i}|^{q} \Biggr)^{\frac {p}{q}} \Biggl(\sum_{i=1}^{n}1 \Biggr)^{1-\frac{p}{q}}=O(n). $$

Obviously, for $1\le i\le n$, it has

3.10 $$\begin{aligned} E \bigl(Y_{ni}^{2}|\mathcal{G}_{i-1} \bigr) =&E \bigl[A_{i}^{2}X_{i}^{2}I\bigl( \vert X_{i} \vert \le c_{n}\bigr)|\mathcal{G}_{i-1} \bigr] \\ &{}- \bigl[E\bigl(A_{i}X_{i}I\bigl( \vert X_{i} \vert \le c_{n}\bigr)|\mathcal{G}_{i-1} \bigr) \bigr]^{2} \\ \le&E \bigl[A_{i}^{2}X_{i}^{2}I\bigl( \vert X_{i} \vert \le c_{n}\bigr)|\mathcal{G}_{i-1} \bigr] \\ \le&EA_{i}^{2}E \bigl(X_{i}^{2}| \mathcal{G}_{i-1} \bigr),\quad \mbox{a.s.} \end{aligned}$$

Combining (3.9) and (2.2), we obtain that

3.11 $$\begin{aligned} J_{2} \le&\sum_{n=1}^{\infty}\frac{b_{n}l(n)}{c_{n}^{q}}E \Biggl(\sum_{i=1}^{n}EA_{i}^{2}E \bigl(X_{i}^{2}|\mathcal{G}_{i-1} \bigr) \Biggr)^{q/2} \\ \le&\sum_{n=1}^{\infty}\frac{b_{n}l(n)}{c_{n}^{q}} \Biggl(\sum_{i=1}^{n}EA_{i}^{2} \Biggr)^{q/2}E \Bigl(\sup_{i\ge1}E \bigl(X_{i}^{2}| \mathcal{G}_{i-1} \bigr) \Bigr)^{q/2} \\ \le&C\sum_{n=1}^{\infty}\frac{n^{q/2}b_{n}l(n)}{c_{n}^{q}}< \infty. \end{aligned}$$

By (3.1) and (3.3)–(3.11), we can get (2.5). This completes the proof of Theorem 2.1.

### Proof of Theorem 2.2

As the proof of Theorem 2.1,

$$A_{i}X_{i}=A_{i}X_{i}I\bigl( \vert X_{i} \vert >c_{n}\bigr)+Y_{ni}+E \bigl[A_{i}X_{i}I\bigl( \vert X_{i} \vert \le c_{n}\bigr)|\mathcal{G}_{i-1}\bigr], $$

where $Y_{ni}=A_{i}X_{i}I(|X_{i}|\le c_{n})-E[A_{i}X_{i}I(|X_{i}|\le c_{n})|\mathcal {G}_{i-1}]$, $i=1, 2, \ldots $ . By Lemma 3.3 with $a=c_{n}$, we have

3.12 $$\begin{aligned}& \sum_{n=1}^{\infty}b_{n}l(n)E \Biggl(\max_{1\le j\le n} \Biggl\vert \sum _{i=1}^{j}A_{i}X_{i} \Biggr\vert -\epsilon c_{n} \Biggr)^{+} \\& \quad \le C\sum_{n=1}^{\infty}\frac{b_{n}l(n)}{c_{n}^{q-1}}E \Biggl(\max_{1\le j\le n} \Biggl\vert \sum _{i=1}^{j}Y_{ni} \Biggr\vert ^{q} \Biggr) \\& \qquad {} +\sum_{n=1}^{\infty}b_{n}l(n)E \Biggl(\max_{1\le j\le n} \Biggl\vert \sum _{i=1}^{j} \bigl[A_{i}X_{i}I \bigl( \vert X_{i} \vert >c_{n}\bigr)+E \bigl(A_{i}X_{i}I\bigl( \vert X_{i} \vert \le c_{n}\bigr)|\mathcal {G}_{i-1}\bigr) \bigr] \Biggr\vert \Biggr) \\& \quad \le \sum_{n=1}^{\infty}b_{n}l(n)E \Biggl(\max_{1\le j\le n} \Biggl\vert \sum _{i=1}^{j}A_{i}X_{i}I \bigl( \vert X_{i} \vert >c_{n}\bigr) \Biggr\vert \Biggr) \\& \qquad {} +\sum_{n=1}^{\infty}b_{n}l(n)E \Biggl(\max_{1\le j\le n} \Biggl\vert \sum _{i=1}^{j}E\bigl[A_{i}X_{i}I \bigl( \vert X_{i} \vert \le c_{n}\bigr)| \mathcal{G}_{i-1}\bigr] \Biggr\vert \Biggr) \\& \qquad {} +C\sum_{n=1}^{\infty}\frac{b_{n}l(n)}{c_{n}^{q-1}}E \Biggl(\max_{1\le j\le n} \Biggl\vert \sum _{i=1}^{j}Y_{ni} \Biggr\vert ^{q} \Biggr) \\& \quad = :H_{1}+H_{2}+H_{3}. \end{aligned}$$

To prove (2.11), it is enough to show $H_{1}<\infty$, $H_{2}<\infty $, and $H_{3}<\infty$.

Since $\{A_{n}, n\ge1\}$ is independent of $\{X_{n}, n\ge1\}$, we have by Lemma 3.2, (3.2), (2.7), and (2.10) that

3.13 $$\begin{aligned} H_{1} \le&\sum_{n=1}^{\infty}b_{n}l(n)\sum_{i=1}^{n}E \vert A_{i} \vert E\bigl[ \vert X_{i} \vert I\bigl( \vert X_{i} \vert >c_{n}\bigr)\bigr] \\ \le&C\sum_{n=1}^{\infty}nb_{n}l(n)E \bigl[ \vert X \vert I\bigl( \vert X \vert >c_{n}\bigr)\bigr] \\ =&C\sum_{n=1}^{\infty}nb_{n}l(n) \sum_{m=n}^{\infty}E\bigl[ \vert X \vert I \bigl(c_{m}< \vert X \vert \le c_{m+1}\bigr)\bigr] \\ =&C\sum_{m=1}^{\infty}E\bigl[ \vert X \vert I\bigl(c_{m}< \vert X \vert \le c_{m+1}\bigr)\bigr] \sum_{n=1}^{m}nb_{n}l(n) \\ \le&C\sum_{m=1}^{\infty}E\bigl[ \vert X \vert I\bigl(c_{m}< \vert X \vert \le c_{m+1}\bigr) \bigr]c_{m}^{p-1}l\bigl(c_{m}^{1/\alpha}\bigr) \\ \le&CE \vert X \vert ^{p}l\bigl( \vert X \vert ^{1/\alpha}\bigr)< \infty. \end{aligned}$$

For $H_{2}$, by a similar proof of (3.4), we have $E(A_{n}X_{n}|\mathcal{G}_{n-1})=0$ a.s., $n\ge1$. Combining with (3.13), we get

3.14 $$\begin{aligned} H_{2} =&\sum_{n=1}^{\infty}b_{n}l(n)E \Biggl(\max_{1\le j\le n} \Biggl\vert \sum _{i=1}^{j}E\bigl[A_{i}X_{i}I \bigl( \vert X_{i} \vert \le c_{n}\bigr)| \mathcal{G}_{i-1}\bigr] \Biggr\vert \Biggr) \\ =&\sum_{n=1}^{\infty}b_{n}l(n)E \Biggl(\max_{1\le j\le n} \Biggl\vert \sum _{i=1}^{j}E\bigl[A_{i}X_{i}I \bigl( \vert X_{i} \vert >c_{n}\bigr)| \mathcal{G}_{i-1}\bigr] \Biggr\vert \Biggr) \\ \le&\sum_{n=1}^{\infty}b_{n}l(n) \sum_{i=1}^{n}E|A_{i}|E\bigl[ \vert X_{i} \vert I\bigl( \vert X_{i} \vert >c_{n}\bigr)\bigr] \\ \le&E|X|^{p}l\bigl(|X|^{1/\alpha}\bigr)< \infty. \end{aligned}$$

Next, from a similar proof of Theorem 2.1 (see (3.5)), we turn to prove $H_{3}<\infty$.

3.15 $$\begin{aligned} H_{3} =&C\sum_{n=1}^{\infty}\frac{b_{n}l(n)}{c_{n}^{q-1}}E \Biggl(\max_{1\le j\le n} \Biggl\vert \sum _{i=1}^{j}Y_{ni} \Biggr\vert \Biggr)^{q} \\ \le&C\sum_{n=1}^{\infty}\frac{b_{n}l(n)}{c_{n}^{q-1}}\sum _{i=1}^{n}E|Y_{ni}|^{q}+C \sum_{n=1}^{\infty}\frac {b_{n}l(n)}{c_{n}^{q-1}}E \Biggl( \sum_{i=1}^{n}E \bigl(Y_{ni}^{2}| \mathcal {G}_{i-1} \bigr) \Biggr)^{q/2} \\ =:&H_{31}+H_{32}. \end{aligned}$$

For $H_{31}$, by $C_{r}$-inequality, Lemma 3.2, and (2.9), we have

3.16 $$\begin{aligned} H_{31} \le&C\sum_{n=1}^{\infty}\frac{b_{n}l(n)}{c_{n}^{q-1}}\sum_{i=1}^{n}E \vert A_{i} \vert ^{q}E \bigl[ \vert X_{i} \vert ^{q}I\bigl( \vert X_{i} \vert \le c_{n}\bigr) \bigr] \\ \le&C\sum_{n=1}^{\infty}\frac{b_{n}l(n)}{c_{n}^{q-1}}\sum _{i=1}^{n}E \vert A_{i} \vert ^{q}E \bigl[ \vert X \vert ^{q}I\bigl( \vert X \vert \le c_{n}\bigr) \bigr] \\ &{}+C\sum_{n=1}^{\infty}nb_{n}l(n)c_{n}P\bigl( \vert X \vert >c_{n} \bigr) \\ \le&C\sum_{n=1}^{\infty}\frac{nb_{n}l(n)}{c_{n}^{q-1}}E \bigl[ \vert X \vert ^{q}I\bigl( \vert X \vert \le c_{n}\bigr) \bigr] \\ &{}+C\sum_{n=1}^{\infty}nb_{n}l(n)E\bigl[ \vert X \vert I\bigl( \vert X \vert >c_{n}\bigr)\bigr] \\ =:&C\widehat{H_{31}}+C\widehat{H_{32}}. \end{aligned}$$

From the condition $q>\max\{2, p\}$, (2.7), and (2.10), we get

3.17 $$\begin{aligned} \widehat{H_{31}} =&\sum_{n=1}^{\infty}\frac{nb_{n}l(n)}{c_{n}^{q-1}}\sum_{m=1}^{n}E \bigl[ \vert X \vert ^{q}I\bigl(c_{m-1}< \vert X \vert \le c_{m}\bigr) \bigr] \\ =&\sum_{m=1}^{\infty}E \bigl[ \vert X \vert ^{q}I\bigl(c_{m-1}< \vert X \vert \le c_{m}\bigr) \bigr]\sum_{n=m}^{\infty}\frac{nb_{n}l(n)}{c_{n}^{q-1}} \\ \le&C\sum_{m=1}^{\infty}E \bigl[ \vert X \vert ^{q}I\bigl(c_{m-1}< \vert X \vert \le c_{m}\bigr) \bigr]c_{m}^{p-q}l\bigl(c_{m}^{1/\alpha} \bigr) \\ \le&CE \vert X \vert ^{p}l\bigl( \vert X \vert ^{1/\alpha}\bigr)< \infty. \end{aligned}$$

By the proof of (3.13), it follows

3.18 $$ \widehat{H_{32}}=\sum_{n=1}^{\infty}nb_{n}l(n)E\bigl[ \vert X \vert I\bigl( \vert X \vert >c_{n}\bigr)\bigr]\le CE \vert X \vert ^{p}l\bigl( \vert X \vert ^{1/\alpha}\bigr)< \infty. $$

For $H_{32}$, from a similar proof of Theorem 2.1 (see (3.9)–(3.11)), combining (3.10), (3.9), and (2.8), we get

3.19 $$\begin{aligned} H_{32} \le&\sum_{n=1}^{\infty}\frac{b_{n}l(n)}{c_{n}^{q-1}}E \Biggl(\sum_{i=1}^{n}EA_{i}^{2}E \bigl(X_{i}^{2}|\mathcal{G}_{i-1} \bigr) \Biggr)^{q/2} \\ \le&\sum_{n=1}^{\infty}\frac{b_{n}l(n)}{c_{n}^{q-1}} \Biggl(\sum_{i=1}^{n}EA_{i}^{2} \Biggr)^{q/2}E \Bigl(\sup_{i\ge1}E \bigl(X_{i}^{2}| \mathcal{G}_{i-1} \bigr) \Bigr)^{q/2} \\ \le&C\sum_{n=1}^{\infty}\frac{n^{q/2}b_{n}l(n)}{c_{n}^{q-1}}< \infty. \end{aligned}$$

Therefore, we can get (2.11) by (3.12)–(3.19). This completes the proof of Theorem 2.2.

### Proof of Theorem 2.3

By a similar proof of Theorem 2.2, we take $q=2$. It is enough to prove $H_{1}<\infty$, $H_{2}<\infty$, and $H_{3}<\infty$. Combining (3.13) with conditions (2.12), (2.13), we have

3.20 $$\begin{aligned} H_{1} =&\sum_{n=1}^{\infty}b_{n}l(n)E \Biggl(\max_{1\le j\le n} \Biggl\vert \sum _{i=1}^{j}A_{i}X_{i}I \bigl( \vert X_{i} \vert >c_{n}\bigr) \Biggr\vert \Biggr) \\ \le&\sum_{n=1}^{\infty}b_{n}l(n) \sum_{i=1}^{n}E \vert A_{i} \vert E\bigl[ \vert X_{i} \vert I\bigl( \vert X_{i} \vert >c_{n}\bigr)\bigr] \\ \le&C\sum_{m=1}^{\infty}E\bigl[ \vert X \vert I\bigl(c_{m}< \vert X \vert \le c_{m+1}\bigr)\bigr] \sum_{n=1}^{m}nb_{n}l(n) \\ \le&C\sum_{m=1}^{\infty}E\bigl[ \vert X \vert I\bigl(c_{m}< \vert X \vert \le c_{m+1}\bigr) \bigr](\log c_{m})l\bigl(c_{m}^{1/\alpha}\bigr) \\ \le&CE \bigl[ \vert X \vert \bigl(\log \vert X \vert \bigr)l\bigl( \vert X \vert ^{1/\alpha}\bigr) \bigr]< \infty. \end{aligned}$$

By the proof of (3.14) and (3.20), we get

3.21 $$\begin{aligned} H_{2} =&\sum_{n=1}^{\infty}b_{n}l(n)E \Biggl(\max_{1\le j\le n} \Biggl\vert \sum _{i=1}^{j}E\bigl[A_{i}X_{i}I \bigl( \vert X_{i} \vert \le c_{n}\bigr)| \mathcal{G}_{i-1}\bigr] \Biggr\vert \Biggr) \\ \le&\sum_{n=1}^{\infty}b_{n}l(n) \sum_{i=1}^{n}E \vert A_{i} \vert E\bigl[ \vert X_{i} \vert I\bigl( \vert X_{i} \vert >c_{n}\bigr)\bigr] \\ \le&E \bigl[ \vert X \vert \bigl(\log \vert X \vert \bigr)l\bigl( \vert X \vert ^{1/\alpha}\bigr) \bigr]< \infty. \end{aligned}$$

For $H_{3}$, from a similar proof of Theorem 2.1 (see (3.5) for $q=2$), we have

$$\begin{aligned} H_{3}&=C\sum_{n=1}^{\infty}\frac{b_{n}l(n)}{c_{n}}E \Biggl(\max_{1\le j\le n} \Biggl\vert \sum _{i=1}^{j}Y_{ni} \Biggr\vert ^{2} \Biggr) \\ &=C\sum_{n=1}^{\infty}\frac{b_{n}l(n)}{c_{n}}E \Biggl\{ E \Biggl(\max_{1\le j\le n} \Biggl\vert \sum _{i=1}^{j}\bigl[a_{i}X_{ni}-E(a_{i}X_{ni}| \mathcal {G}_{i-1})\bigr] \Biggr\vert ^{2} \Biggr) \Big|A_{1}=a_{1}, \ldots, A_{n}=a_{n} \Biggr\} \\ &\le C\sum_{n=1}^{\infty}\frac{b_{n}l(n)}{c_{n}}E \Biggl\{ \Biggl(\sum_{i=1}^{n}E| \hat{Y}_{ni}|^{2} \Biggr)\Big|A_{1}=a_{1}, \ldots, A_{n}=a_{n} \Biggr\} \\ &=C\sum_{n=1}^{\infty}\frac{b_{n}l(n)}{c_{n}}\sum _{i=1}^{n}E|Y_{ni}|^{2}. \end{aligned}$$

Then, according to (2.12) and (3.20), we can get

3.22 $$\begin{aligned} H_{3} \le&C\sum_{n=1}^{\infty}\frac{b_{n}l(n)}{c_{n}}\sum_{i=1}^{n}E \vert A_{i} \vert ^{2}E \bigl[ \vert X_{i} \vert ^{2}I\bigl( \vert X_{i} \vert \le c_{n}\bigr) \bigr] \\ \le&C\sum_{n=1}^{\infty}\frac{nb_{n}l(n)}{c_{n}}\sum _{i=1}^{n}E \bigl[ \vert X \vert ^{2}I\bigl( \vert X \vert \le c_{n}\bigr) \bigr]+C\sum _{n=1}^{\infty}nb_{n}l(n)c_{n}P \bigl( \vert X \vert >c_{n}\bigr) \\ \le&C\sum_{n=1}^{\infty}\frac{nb_{n}l(n)}{c_{n}}\sum _{m=1}^{n}E \bigl[ \vert X \vert ^{q}I\bigl(c_{m-1}< \vert X \vert \le c_{m} \bigr) \bigr] \\ &{}+C\sum_{n=1}^{\infty}nb_{n}l(n)E\bigl[ \vert X \vert I\bigl( \vert X \vert >c_{n}\bigr)\bigr] \\ =&\sum_{m=1}^{\infty}E \bigl[ \vert X \vert ^{q}I\bigl(c_{m-1}< \vert X \vert \le c_{m}\bigr) \bigr]\sum_{n=m}^{\infty}\frac{nb_{n}l(n)}{c_{n}} \\ &{}+CE \bigl[ \vert X \vert \bigl(\log \vert X \vert \bigr)l\bigl( \vert X \vert ^{1/\alpha}\bigr) \bigr] \\ \le&C\sum_{m=1}^{\infty}E \bigl[ \vert X \vert ^{q}I\bigl(c_{m-1}< \vert X \vert \le c_{m}\bigr) \bigr]c_{m}^{1-q}l\bigl(c_{m}^{1/\alpha} \bigr)+C< \infty. \end{aligned}$$

Hence, the desired result follows from (3.20)–(3.22). This completes the proof of Theorem 2.3.
